# GSTP1 as a novel target in radiation induced lung injury

**DOI:** 10.1186/s12967-021-02978-0

**Published:** 2021-07-08

**Authors:** Xiao Lei, Lehui Du, Wei Yu, Yao Wang, Na Ma, Baolin Qu

**Affiliations:** Department of Radiation Oncology, The Fifth Medical Center of the Chinese PLA General Hospital , Beijing, China

**Keywords:** Radiation induced lung injury, GSTP1, Inflammatory response, Fibrosis

## Abstract

The glutathione S-transferase P1(GSTP1) is an isoenzyme in the glutathione-S transferases (GSTs) enzyme system, which is the most abundant GSTs expressed in adult lungs. Recent research shows that GSTP1 is closely related to the regulation of cell oxidative stress, inhibition of cell apoptosis and promotion of cytotoxic metabolism. Interestingly, there is evidence that GSTP1 single nucleotide polymorphisms (SNP) 105Ile/Val related to the risk of radiation induced lung injury (RILI) development, which strongly suggests that GSTP1 is closely associated with the occurrence and development of RILI. In this review, we discuss our understanding of the role of GSTP1 in RILI and its possible mechanism.

## Background

Radiotherapy is one of the main treatments for malignant tumors, it brings great benefits for tumor patients, which could significantly improve the quality of the life and prolong the survival [1]. However, Radiation induced lung injury (RILI) is the main dose-limiting toxicity of thoracic radiation, which seriously affects the implementation of radiotherapy plan and patients’ prognosis [2, 3]. Although the rapid developments of medical imaging, computer technologies and radiotherapy equipment assist in the transformation of radiotherapy from traditional radiotherapy to precision radiotherapy, RILI remains to be the most common side effect for lung cancer patients, with 5%–36% of patients experiencing symptomatic (grade 2–5) RILI [3–5]. On the one hand, the advanced radiotherapy technology cannot avoid the direct damage of radiation to the normal tissue around tumor completely. On the other hand, the biological off-target effect caused by radiation causes the normal tissue which is not involved in the radiation area to be also affected [6, 7]. Besides lung tissue is one of the most radiosensitive organs in human, therefore, how to reduce and prevent RILI is thus vital. At present, the main mechanism of RILI is still unclear, and there is no clinically effective drug for it. Nowadays the clinical treatment of RILI is mainly based on the use of glucocorticoids, combined with symptomatic treatment such as antibiotics, relieving cough, reducing phlegm and relieving asthma drugs, but none of them are specific treatment [8–10]. Therefore, it is urgent to explore the mechanism of RILI and find effective therapeutic strategy. Interestingly, recent research shows that GSTP1 SNP 105Ile/Val related to the risk of RILI development, which strongly suggests that GSTP1 is closely associated with the occurrence and development of RILI [11, 12]. GSTP1 SNP 105Ile/Val is a GSTP1 variant which bearing a single nucleotide polymorphism in exon 5 (Ile105Val, rs1695), its enzymatic activity is reduced by 50%–70% compared to the wild-type GSTP1 [11, 13]. Combined with the multiple functions of GSTP1 [14–16], we speculate that targeting GSTP1 may be a new more effective treatment method for RILI. In this review, we discuss current research progress of the potential role of GSTP1 in RILI and its mechanism.

### The potential role and its mechanisms of GSTP1 in radiation induced lung injury

GSTP1 is the most abundant protein subtype in glutathione S transferase family, which mainly contains α, μ, π, θ subtypes. The GTSP1 gene is located on chromosome 11q13, which consists of nine exons and 3.2 kb in length [17, 18]. GSTP1 can catalyze the combination of glutathione (GSH) with various electrophilic and hydrophobic substances to form water-soluble compounds to be discharged from body, thereby reducing the damage of intracellular biological macromolecules [16, 19]. It is a dimeric protein composed of two same subunits with a molecular weight of 22.5 kDa [20]. Recent research shows that GSTP1 plays a vital role in maintaining the balance of cell oxidation and regulating cell proliferation and apoptosis [21–23]. Furthermore, clinical data finds that patients with GSTP1 SNP 105Ile/Val had significantly higher risk of radiation pneumonitis (RP) of grade ≥ 2 (P = 0.001), which strongly indicates that GSTP1 is closely associated with the occurrence and development of RILI [11]. Although there is no experimental study on the effect of GSTP1 on RILI, we consider that GSTP1 has the potential role to protect against RILI combining with the anti-oxidation and anti-apoptosis functions of GSTP1. The potential mechanisms of GSTP1 in RILI as follows.

### GSTP1 is involved in the regulation of inflammatory response

Multiple studies have shown that GSTP1 can inhibit inflammation through multiple pathways [24–27]. The first and most important mode is via inhibiting reactive oxygen species (ROS) pathway [24, 28]. As we mentioned before, GSTP1 belongs to GST family, which can catalyze intracellular detoxification reactions so that plays role in buffering ROS [29]. Kecheng Lei shows that MNPC, a small molecule NQO1 and GSTP1 dual inhibitor, can reduce ROS reaction via inhibiting both NADPH quinone oxidoreductase 1 (NQO1) and GSTP1, leading to apoptosis and mitigated glioblastoma (GBM) cell proliferation [24]. Other studies provide evidence that GSTP1 is phase-II detoxification enzymes that reduce quinones directly to hydroquinones, eliminating the formation of ROS produced by redox cycling [30, 31]. The second mode is via inhibiting LPS-stimulated MAPKs (mitogen-activated protein kinases) [32–34]. Lan Luo shows that GSTP1 can suppress lipopolysaccharides (LPS) induced excessive production of pro-inflammatory factors by inhibiting LPS-stimulated MAPKs as well as NF-kappaB activation. Besides, GSTP1 can prevent LPS-induced TNF-alpha, IL-1beta, MCP-1 and NO production. Through the above method, GSTP1 can alleviate LPS-induced acute lung injury [33, 35]. Recent research also shows that GSTP1 can inhibit LPS-induced inflammatory response through regulating autophagy [34]. The third mode is via regulating mitochondrial energetics and cellular metabolism [36]. Marianne E Fletcher finds that inhibiting GSTP1 can aggravate pulmonary edema, inflammatory cell infiltration and other inflammatory reactions through the third mode [37]. In summary, GSTP1 could regulate inflammation response through the above three modes.

### GSTP1 is related to radiation induced fibrosis

RILI includes radiation pneumonitis and radiation induced pulmonary fibrosis. It’s the consequence for acute and late effects of radiation. Radiation pneumonitis occurs in 6 months after radiation while radiation induced pulmonary fibrosis occurs > 6 months [3, 38]. Recent clinical researches show that GSTP1 is associated with radiation induced fibrosis to various tissues [39, 40]. Hege Edvardsen followed up 92 breast cancer survivors previously treated with hypofractionated radiation therapy, did the SNPs genotype and found GSTP1 was highly associated with subcutaneous fibrosis and lung fibrosis, which might be due to participate in maintenance of the intracellular redox balance [41]. Fangming Kan utilized RNA sequencing and Gene Ontology (GO) analysis on mice liver tissue infected with Hepatitis B virus (HBV), then he discovered GSTP1 was one of the key proteins involved in HBV-related liver fibrosis [42]. Terrazzino did the research with 257 breast cancer patients who underwent surgery plus adjuvant radiotherapy and found GSTP1 Ile105Val was significantly associated with the risk of Grade 2–3 radiation-induced fibrosis, especially in skin fibrosis [43]. Lehui Du recruited a total of 149 lung cancer patients who had received intensity modulated radiation therapy (IMRT) and did blood samples DNA extraction and genotyping, then they found an association between GSTP1 SNP 105Ile/Val and risk of RP development, which suggests the potential use of this genetic polymorphism as a predictor of RP [11]. In summary, GSTP1 involves in various tissues’ radiation induced fibrosis, which indicates GSTP1 plays an important role in RILI.

### GSTP1 regulates radiation induced cell apoptosis

Plenty of studies have shown that GSTP1 can regulate radiation induced apoptosis through multiple pathways [44–46]. T Wang discovered that GSTP1 Inhibited C-Jun N-Terminal Kinase (Jnk1) signaling through interaction with the C Terminus [47], while Ruscoe JE showed that GSTP1 could inhibit radiation induced cell apoptosis via GSTP1-Jnk1 pathway [48]. GSTP1 also could inhibit the interaction between tumor necrosis factor-related receptor 2 (TRAF2) and apoptosis signal-regulating kinase 1 (ASK1) by binding to TRAF2, negatively regulated the ASK1–MEK–JNK/p38 pathway induced by tumor necrosis factor α (TNFα), which inhibited cell apoptosis [30]. However, Guyue Liu showed that GSTP1 could induce apoptosis in malignant hematologic cells via downregulation of myeloid cell leukemia-1 (Mcl-1) and cellular FLICE (FADD-like IL-1β-converting enzyme)-inhibitory protein (c-FLIP) [49]. GSTP1 also could induce apoptosis through the Fas-ASK1-JNK/p38 pathway by amplifying Fas ligand [50]. In summary, the mechanism of GSTP1 regulating cell apoptosis and proliferation is thus complicated, which need to further explore.

### Perspective

**Fig. 1:**
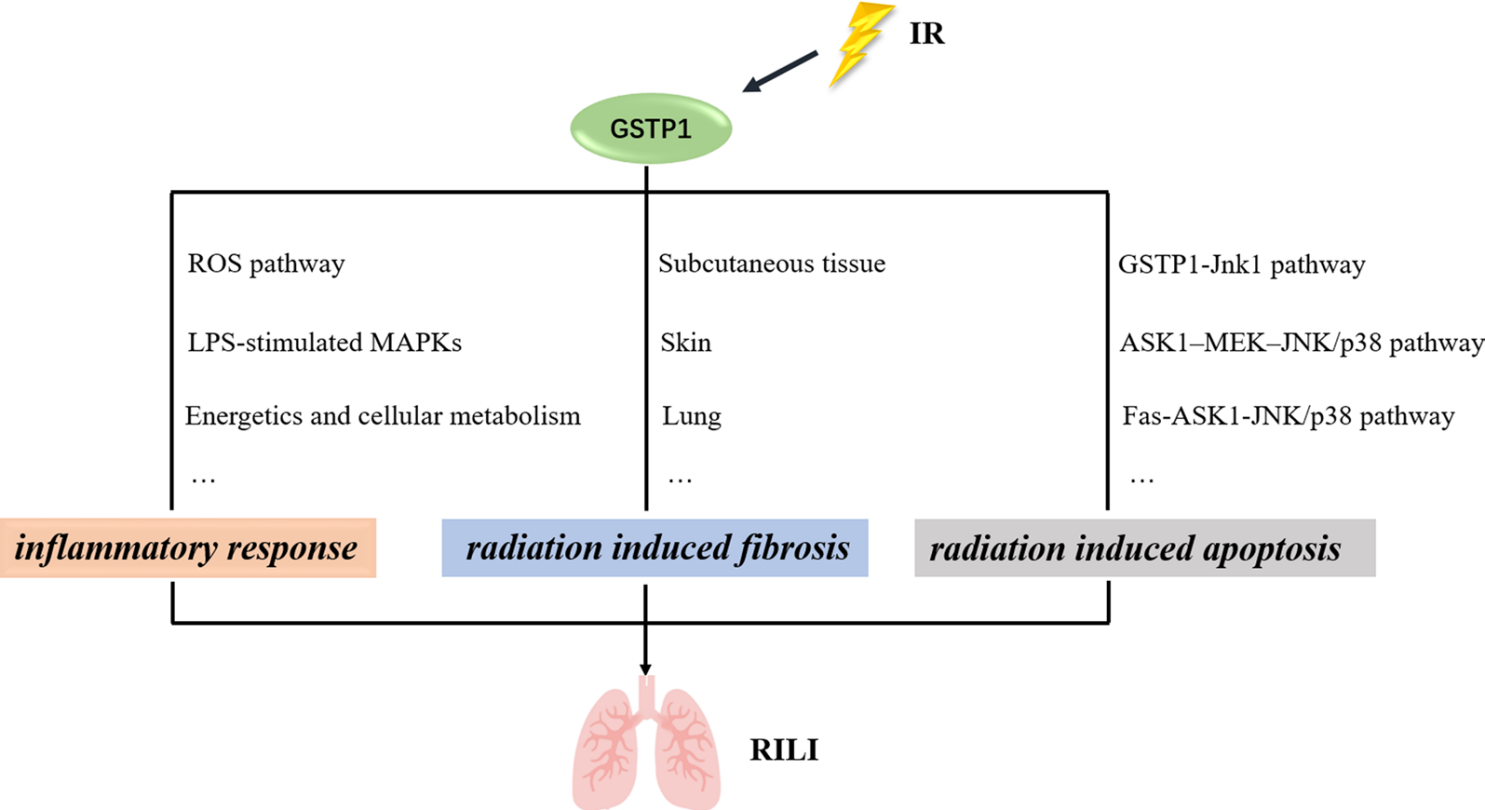
Potential mechanisms of GSTP1 in RILI

Although the development of medical technology has greatly alleviated the occurrence of adverse events of radiotherapy, lung tissue, as one of the most radiosensitive organs, is impossible to avoid damage to patients with chest tumors after radiotherapy [51–54]. Therefore, RILI is still the most common complication after radiotherapy for patients with chest tumors [3]. RILI includes radiation pneumonitis and radiation induced pulmonary fibrosis, the pathogenesis of which is still not completely clear [55]. Mainstream researches show that the mechanism of RILI may be because ionizing radiation can directly cause DNA damage and a large number of free radicals in lung tissue cells, which can mediate and amplify the damage of alveolar epithelial cells and vascular endothelial cells by promoting oxidative stress, vascular damage and inflammatory response, further leading to RILI [56–58]. Nowadays there is no specific therapeutic drug for radiation-induced lung injury, only glucocorticoids are used in the treatment of RILI, but it’s not specific treatment [10]. Therefore, there is an urgent need to find a strategy to prevent or treat RILI in clinic. GSTP1 is a protein subtype in glutathione S transferase family, which shows great role in maintaining the balance of cell oxidation and regulating cell proliferation and apoptosis [23, 59]. Although there is no experimental research indicating GSTP1 plays crucial role in RILI, Du’s research presents GSTP1 SNP 105Ile/Val is a vital predictor of RILI, which raise our interest that GSTP1 may have the potential role to protect against RILI [11]. The potential mechanisms of GSTP1 protecting against RILI have already present in this review, which means that GSTP1 may be the significant target for clinical protection of RILI (Fig. 1). However, recent research shows that GSTP1 is closely related to tumor occurrence and prognosis [60–63]. GSTP1 is highly expressed in a variety of tumor tissues and can be used as a biomarker in tumors [64–66]. Therefore, research needs to further explore the role of GSTP1 in tumor tissue after radiotherapy, so that clinic can make individual treatment for patients to reduce the occurrence of RILI.

## Conclusion

All in all, GSTP1 is an important predictor of RILI and may play vital role in prevention of RILI. The potential mechanisms of GSTP1 in RILI include inflammatory response, radiation induced fibrosis and cell apoptosis. However, there is still no clear experiment to show the role and mechanism of GSTP1 in RILI. This is a research idea we provide for everyone, which could make better individualized treatment strategies for the clinic. Furthermore, although related studies have shown that GSTP1 is closely related to the occurrence and development of tumors, there is no research showing the role of GSTP1 in tumor tissues after radiotherapy. Till now, there are several evidences showing that targeting GSTP1 can have a synergistic effect with chemotherapy drugs, but no relevant study shown between GSTP1 and chemotherapy-induced lung injury [66–68]. If the changes in the expression or activity of GSTP1 protein could be controlled to increase the destructive effect of radiotherapy on tumor tissues, while reducing radiation damage to the normal lung tissue, it will have extremely important medical significance for the treatment and prognosis of patients with thoracic tumors. This is the core purpose of this review.

## Data Availability

The datasets are available under reasonable request.
